# Alpha and gamma mangostins inhibit wild-type B SARS-CoV-2 more effectively than the SARS-CoV-2 variants and the major target is unlikely the 3C-like protease

**DOI:** 10.1016/j.heliyon.2024.e31987

**Published:** 2024-05-27

**Authors:** Aphinya Suroengrit, Van Cao, Patcharin Wilasluck, Peerapon Deetanya, Kittikhun Wangkanont, Kowit Hengphasatporn, Ryuhei Harada, Supakarn Chamni, Asada Leelahavanichkul, Yasuteru Shigeta, Thanyada Rungrotmongkol, Supot Hannongbua, Warinthorn Chavasiri, Supaporn Wacharapluesadee, Eakachai Prompetchara, Siwaporn Boonyasuppayakorn

**Affiliations:** a Center of Excellence in Applied Medical Virology, Department of Microbiology, Faculty of Medicine, Chulalongkorn University, Bangkok, 10330, Thailand; b Research Affairs, Faculty of Medicine, Chulalongkorn University, Bangkok, 10330, Thailand; c Interdisciplinary Program in Microbiology, Graduate School, Chulalongkorn University, Bangkok, 10330, Thailand; d DaNang University of Medical Technology and Pharmacy, DaNang, 50200, Viet Nam; e Center of Excellence for Molecular Biology and Genomics of Shrimp, Department of Biochemistry, Faculty of Science, Chulalongkorn University, Bangkok, 10330, Thailand; f Center of Excellence in Molecular Crop, Department of Biochemistry, Faculty of Science, Chulalongkorn University, Bangkok, 10330, Thailand; g Center for Computational Sciences, University of Tsukuba, 1-1-1 Tennodai, Tsukuba, Ibaraki, 305-8577, Japan; h Department of Pharmacognosy and Pharmaceutical Botany, Faculty of Pharmaceutical Sciences, Chulalongkorn University, Bangkok, 10330, Thailand; i Center of Excellence in Natural Products and Nanoparticles (NP2), Chulalongkorn University, Bangkok, 10330, Thailand; j Center of Excellence in Translational Research in Inflammation and Immunology (CETRII), Department of Microbiology, Faculty of Medicine, Chulalongkorn University, Bangkok, 10330, Thailand; k Program in Bioinformatics and Computational Biology, Graduate School, Chulalongkorn University, Bangkok, 10330, Thailand; l Center of Excellence in Structural and Computational Biology, Department of Biochemistry, Faculty of Science, Chulalongkorn University, Bangkok, 10330, Thailand; m Center of Excellence in Natural Products Chemistry, Department of Chemistry, Faculty of Science, Chulalongkorn University, Bangkok, 10330, Thailand; n Thai Red Cross Emerging Infectious Diseases Clinical Center, King Chulalongkorn Memorial Hospital, Bangkok, 10330, Thailand; o Center of Excellence in Vaccine Research and Development, Chulalongkorn University (Chula-VRC), Bangkok, 10330, Thailand; p Department of Laboratory Medicine, Faculty of Medicine, Chulalongkorn University, Bangkok, Thailand

**Keywords:** Alpha mangostin, Gamma mangostin, 3C-like protease, Antiviral drug discovery, SARS-CoV-2 drugs, COVID-19 drug, Target identification

## Abstract

**Background:**

Anti-SARS-CoV-2 and immunomodulatory drugs are important for treating clinically severe patients with respiratory distress symptoms. Alpha- and gamma-mangostins (AM and GM) were previously reported as potential 3C-like protease (3CL^pro^) and Angiotensin-converting enzyme receptor 2 (ACE2)-binding inhibitors *in silico*.

**Objective:**

We aimed to evaluate two active compounds, AM and GM, from *Garcinia mangostana* for their antivirals against SARS-CoV-2 in live virus culture systems and their cytotoxicities using standard methods. Also, we aimed to prove whether 3CL^pro^ and ACE2 neutralization were major targets and explored whether any additional targets existed.

**Methods:**

We tested the translation and replication efficiencies of SARS-CoV-2 in the presence of AM and GM. Initial and subgenomic translations were evaluated by immunofluorescence of SARS-CoV-2 3CL^pro^ and N expressions at 16 h after infection. The viral genome was quantified and compared with the untreated group. We also evaluated the efficacies and cytotoxicities of AM and GM against four strains of SARS-CoV-2 (wild-type B, B.1.167.2, B.1.36.16, and B.1.1.529) in Vero E6 cells. The potential targets were evaluated using cell-based anti-attachment, time-of-drug addition, *in vitro* 3CL^pro^ activities, and ACE2-binding using a surrogated viral neutralization test (sVNT). Moreover, additional targets were explored using combinatorial network-based interactions and Chemical Similarity Ensemble Approach (SEA).

**Results:**

AM and GM reduced SARS-CoV-2 3CL^pro^ and N expressions, suggesting that initial and subgenomic translations were globally inhibited. AM and GM inhibited all strains of SARS-CoV-2 at EC_50_ of 0.70–3.05 μM, in which wild-type B was the most susceptible strain (EC_50_ 0.70–0.79 μM). AM was slightly more efficient in the variants (EC_50_ 0.88–2.41 μM), resulting in higher selectivity indices (SI 3.65–10.05), compared to the GM (EC_50_ 0.94–3.05 μM, SI 1.66–5.40). GM appeared to be more toxic than AM in both Vero E6 and Calu-3 cells. Cell-based anti-attachment and time-of-addition suggested that the potential molecular target could be at the post-infection. 3CL^pro^ activity and ACE2 binding were interfered with in a dose-dependent manner but were insufficient to be a major target. Combinatorial network-based interaction and chemical similarity ensemble approach (SEA) suggested that fatty acid synthase (FASN), which was critical for SARS-CoV-2 replication, could be a target of AM and GM.

**Conclusion:**

AM and GM inhibited SARS-CoV-2 with the highest potency at the wild-type B and the lowest at the B.1.1.529. Multiple targets were expected to integratively inhibit viral replication in cell-based system.

## 1 Introduction

The severe acute respiratory syndrome coronavirus 2 (SARS-CoV-2) causes the most recent global pandemic. The vulnerable group is still at risk for severities despite adequate vaccination. Antiviral and immunomodulatory drugs are still essential to treat vulnerable patients. The virus is a member of the family *Coronaviridae*, genus beta-coronavirus, containing a 29.8–29.9 kb positive-sense RNA genome [1,2]. The virus enters cells through receptor-mediated endocytosis and pH-dependent fusion [3]. Initial translation generated polyproteins consisting of 10 and 16 nonstructural proteins (NSP) from the open reading frame (ORF) 1a and 1 ab, respectively. Two viral proteases, a 3C-like protease (3CL^pro^, M^pro^, or NSP5) and a papain-like protease (PL^pro^, or NSP3), are responsible for self-cleaving viral polyproteins. Furthermore, viral proteases cleave inflammatory modulators such as interferon-regulatory factor (IRF) 3, nucleotide-binding leucine-rich repeat and pyrin domain-containing receptor (NLRP) 12, and TGF-beta-activated kinase 1 [4]. Later, structural and accessory proteins were translated from subgenomic RNAs (sgRNA) complementary to the minus-strand replicative intermediate [5]. Translation, replication, and assembly occur in ER-derived double-membrane vesicles derived from the ER. The viral polymerase and proteases are primary drug targets, as they are essential and exclusive to the virus.

Antiviral drugs are prescribed early after infection, while immunomodulators are considered later during an immune phase. Thailand's national guideline for prescribing early, non-severe cases could be any of the following; FDA-approved drugs (e.g. molnupiravir, paxlovid, etc.), or natural products (e.g. *Andrographis paniculata* capsules) in a vulnerable population. Moreover, several reports suggested natural products as potential inhibitors, with one of the most common targets being 3CL^pro^ [[6], [7], [8], [9], [10], [11]]. Alpha, gamma-mangostins (AM and GM), and their derivatives were previously characterized as potential 3CL^pro^ inhibitors based on an *in silico* study [8,[11], [12], [13], [14]]. These compounds were also reported as an angiotensin converting enzyme (ACE)-2 binding inhibitor [15]. Moreover, the *in vivo* toxicity of the crude methanolic mangosteen extract was established at ≤200 mg/kg for a short-term study, and the lethal dose (LD)_50_ was at approximately 1000 mg/kg [16]. The *Garcinia mangostana* crude extracts usually contain AM and GM as the major and minor active ingredients. This study focused on analyzing purified AM and GM as inhibitors of SARS-CoV-2 and three variants in a cell-based system. Various cell-based assays characterized that mangostins mainly inhibited viral translation and replication. However, previously identified 3CL^pro^ and ACE2 were adjunctive targets proven by enzyme activity and surrogate viral neutralization assays. The host-derived FASN predicted by network-based target identification was potentially a target, as this protein was crucial for SARS-CoV-2 replication and was previously identified as an AM target.

## 2 Materials and methods

### 2.1 Cells and virus culture

Vero E6 cells (ATCC, CRL-1587) and Calu-3 cells (HTB-55) were maintained in minimal essential medium (MEM) (Gibco, Langley, OK, USA) supplemented with 10 % fetal bovine serum (Gibco®, Langley, OK, USA), 100 I.U./ml penicillin (Bio Basic Canada, Ontario, CA), and 100 μg/ml streptomycin (Bio Basic Canada, Ontario, CA), 10 mM HEPES (4-(2-hydroxyethyl)-1-piperazineethanesulfonic acid) (Sigma Aldrich, St. Louis, MO, USA), Non-essential amino acid (NEAA) (Gibco, Langley, OK, USA), and sodium pyruvate (Gibco, Langley, OK, USA). Cells were incubated at 37 °C humidified chamber under 5 % CO_2_.

The SARS-CoV-2 wild type B (accession number EPI_ISL_447909), B.1.36.16 (accession number EPI_ISL_3892049.1), B.1.1.529 (EPI_ISL_17735483) and B.1.617.2 (accession number ON381169) were courtesy of Department of Medical Sciences, Ministry of Public health, Thailand, Chula-VRC, AFRIMS, and EIDCC, respectively. All SARS-CoV-2 were propagated in Vero E6 cells with MEM supplemented with 1 % fetal bovine serum, 100 I.U./ml penicillin, and 100 μg/ml streptomycin, 10 mM HEPES, NEAA, and sodium pyruvate at 37 °C humidified chamber under 5 % CO_2_. Virus titers were determined as TCID_50_/ml in confluent cells in 96-well cell culture plates.

All experiments with live SARS-CoV-2 were performed in a certified biosafety level 3 facility of the Research Affairs Medical Research Center (MRC), Faculty of Medicine, Chulalongkorn University. The study was conducted according to the guidelines of the Declaration of Helsinki, and Chulalongkorn University Institutional Biosafety Committee (CU-IBC 003/2021). The Institutional Review Board of Faculty of Medicine, Chulalongkorn University certified the protocol exemption for using a leftover specimen (COE 017/2021, IRB No. 297/64).

### 2.2 Immunofluorescent assays

Vero cells at 1 × 10^4^ cells per well were seeded into 8-well chamber slide in a growth medium and incubated overnight at 37 °C under 5 % CO_2_. Cells were infected with SARS-CoV-2 (wild type B) at the 1000TCID_50_ for 1 h. After infection, cells were washed with phosphate buffer saline (PBS) and incubated with the 4 μM AM or GM in the maintenance medium. Cells were incubated at 37 °C for 16 h, unless indicated otherwise, followed by fixation with 10 % neutral buffered formaldehyde for 1 h. Cells were permeabilized by 0.1 % Triton-X in BSA and blocked with 2 % BSA. The mouse anti-SARS/SARS-CoV-2 nucleocapsid monoclonal antibody (MA5-29981) (ThermoFisher Scientific; Waltham, MA, USA) and FITC-coupled goat-antimouse mouse IgGκ light chain binding protein (*m*-IgGκ BP-FITC, Cat no. sc-516140, Santa Cruz Biotechnology, Dallas, TX, USA) were used to analyze the SARS-CoV-2 nucleocapsid protein. The anti SARS 3Cl protease antibody (200-401-A51) (Rockland, PA, USA) and Goat anti-Rabbit IgG (H + L) Highly Cross-Adsorbed Secondary Antibody, Alexa Fluor™ 568 (ThermoFisher Scientific; Waltham, MA, USA) were used to analyze the SARS-CoV-2 3CLpro. Images were acquired using a laser scanning confocal microscope: LSM 800 (ZEISS, White Plains, NY, USA) and Olympus BX50 fluorescence microscope (Olympus life science, Tokyo, Japan). Results were reported as the proportion of infected cells compared to the DMSO treatment. Errors were indicated by standard deviation (SD) from two independent experiments.

### 2.3 Efficacy study

AM (5 g) was extracted from *Garcinia mangostana* pericarps (200 g), purified by chromatographic method, and identified by spectroscopic analysis as previously described [17,18] whereas GM (Cas. No.31271-07-5) was purchased from Sigma Aldrich® (St. Louis, MO, USA). AM and GM were tested against wild type and three variant strains of SARS-CoV-2. Briefly, Vero E6 cells at 5 × 10^4^ cells per well were seeded into 24-well plate and incubated overnight at 37 °C under 5 % CO_2_. Cells were infected with SARS-CoV-2 at 1000TCID for 1 h. After infection, cells were washed with phosphate buffer saline (PBS) and incubated with 1 ml of maintenance medium. The compounds were prepared at the indicated concentrations in 0.1 % dimethylsulfoxide (Sigma-Aldrich, St-Louis, MO, USA), in the maintenance medium during infection and after infection. Cells were incubated at 37 °C for 72 h under 5 % CO_2_ humidified chamber. Supernatants were collected for analysis of the viral infectivity by TCID_50_/ml (v2.1 - 20-01-2017_MB* by Marco Binder; adapted @ TWC. 5.6, accessed on 16 May 2022). The compound was serially diluted to 6–8 different concentrations and was added to final concentrations into SARS-CoV2-infected cells. DMSO at 0.1 % was used as a vehicle, no inhibition control. Cells were incubated for 72 h and supernatants were collected for subsequent TCID_50_/ml analysis [19,20]. Data were plotted and effective concentration (EC)_50_ values were calculated from nonlinear regression analysis (GraphPad Prism v.10.0, Boston, MA, USA). Results were reported as means and standard deviation (SD) of three independent experiments.

### 2.4 Cytotoxicity in cell-based assay

The cytotoxicity of AM and GM was tested with Vero E6 and Calu-3 cell lines [21]. The compounds were serially diluted to the concentrations respective to the previous description in EC_50_ assay. Cells were incubated for 24 h and were analyzed for their viability using CellTiter 96® AQueous One Solution Cell Proliferation Assay (Promega, Madison, WI, USA). Data were plotted in a nonlinear regression, and the concentration which correlate to 50 % viability (CC_50_) was determined. Results were reported as means and standard error mean (SEM) from three independent experiments.

The real-time cell analysis (RTCA) was performed in Vero E6 cells seeded at 1 × 10^4^ cells per well into a microelectronic 96-well plate (E-plate; Roche, Basel, Switzerland). Cells were incubated overnight at 37 °C under 5 % CO_2_. AM and GM at 1, 5, 10, and 50 μM were added into the cells and monitored by electrical impedance signals [22] every 15 min for 48 h using a real-time cell analyzer (xCELLigence®, Agilent, Santa Clara, CA, USA). The cell-based kinetic profiling was normalized by cells incubated with 0.1 % DMSO in media throughout the experiment. The results were plotted as the means of each time-point performed in duplicates, relative to DMSO.

### 2.5 Anti-attachment study

Vero E6 cells at 5 × 10^4^ cells per well were seeded into 24-well plate and incubated overnight at 37 °C under 5 % CO_2_. Cells were infected with SARS-CoV-2 (wild type B) at the 100TCID_50_ for 1 h. The 4 μM AM or GM compounds were introduced to the system in various conditions as follows; 1) cellular pretreatment (Pr), in which the compounds were added to the cells 1 h prior to the infection, 2) viral neutralization (N), in which the compounds were added to the virus 1 h prior to the infection, 3) co-incubation (C), in which the compounds were added to the cells during infection, 4) post-infection (Po), in which the compounds were added to the cells after the infection and incubated at 37 °C for 24 h. Cells and supernatants were collected for analysis of the viral RNA by RT-qPCR. Results were reported as the proportion of infected cells compared to the DMSO treatment. Remdesivir was a positive inhibitor control. Errors were indicated by standard deviation (SD) from two independent experiments.

Additionally, a cell-based attachment inhibition for immunofluorescence was performed by incubating 4 μM AM and GM with the SARS-CoV-2 during infection for 1 h. Cells were washed and incubated for 6 h before harvest for immunofluorescence study. The anti-SARS 3CL^pro^ antibody and the secondary antibody were previously described in 2.2. Images were acquired using an Olympus BX50 fluorescence microscope (Olympus life science, Tokyo, Japan). Results were reported as the proportion of infected cells compared to the DMSO treatment. Errors were indicated by standard deviation (SD) from two independent experiments.

### 2.6 Time-of-addition study

Vero E6 cells at 5 × 10^4^ cells per well were seeded into 24-well plate and incubated overnight at 37 °C under 5 % CO_2_. Cells were infected with SARS-CoV-2 (wild type B) at the 100TCID_50_ for 1 h. The 4 μM AM or GM compounds were added to the cells after the infection at respective time-points as indicated. Cells were incubated at 37 °C for 24 h and supernatants were collected for analysis of the viral RNA by RT-qPCR. Results were reported as the proportion of infected cells compared to the DMSO treatment. Remdesivir was a positive inhibitor control. Errors were indicated by standard deviation (SD) from two independent experiments.

### 2.7 Reverse transcription and quantitative polymerase chain reaction (RT-qPCR)

The viral RNA in supernatants were extracted by Ribospin vRD II kit (GeneAll Biotechnology, Seoul, Korea) according to manufacturer's protocol. The remaining cells in experimental plates were extracted for viral RNAs using TRIzol reagent (Invitrogen, Waltham, MA, USA) according to the manufacturer's protocol. The samples were loaded into the Direct-zol™ RNA MiniPrep (Zymo research, Irvine, CA, USA) and quantified by Nanodrop spectrophotometry (Eppendorf, Hamburg, Germany). The RT-qPCR was performed with a Real-Time PCR System (Bio-Rad CFX96, Hercules, CA, USA.) with SensiFAST™ SYBR® No-ROX One-Step Kit (Bioline, London, UK) according to the manufacturer's protocol. The primers were N-gene target, forward primer was 5′ CGTTTGGTGGACCCTCAGAT 3′ and reverse primer was 5′ CCCCACTGCGTTCTCCATT 3’ [23]. Each sample was analyzed in duplicated and results were confirmed by two independent experiments.

### 2.8 Protease inhibition assay

The SAR-CoV-2 3CL^pro^ with native termini was produced using the previously reported procedure for SARS-CoV-1 3CL^pro^ [24]. AM and GM were serially diluted in DMSO and freshly prepared for each assay. The protease activity and inhibition assays were performed exactly as described previously [25].

### 2.9 ELISA-based surrogate virus neutralization tests (sVNT)

The commercially available SARS-CoV-2 sVNTs GenScript Cat. No. L00847-A (GenScript Biotech, NJ, USA)) were designed to detect neutralizing antibody between the viral receptor binding domain (RBD) and human angiotensin-converting enzyme 2 (ACE2) receptors. AM or GM at 5, 50, and 100 μM were added to the ACE2-coated ELISA plate and incubated for 1 h before addition of RBD and HRP-RBD according to the manufacturer's protocol. Results were proportionated to the signal from total binding efficiency. Errors were indicated by standard deviation (SD) from two independent experiments.

### 2.10 In vivo toxicity study

All methods were carried out in accordance with relevant guidelines and regulations. All experimental protocols were approved by the Institutional Animal Care and Use Committee of the Faculty of Medicine, Chulalongkorn University, Bangkok, Thailand (certificate number: 017/2565), based on the National Institutes of Health, USA's criteria for the use and treatment of laboratory animals. All methods are reported in accordance with ARRIVE guidelines (https://arriveguidelines.org) for the reporting of animal experiments. The 8-week-old C57BL/6 mice were injected with 0.5 ml of AM and GM at 25 mg/kg diluted with vehicle (5 % DMSO, 33.25 % polyethylene glycol (PEG.)-400, 1.90 % ethanol, and 59.85 % distilled water) or vehicle alone were intraperitoneally administered (n = 5/group) [26]. Statistical analysis was determined using Gpower program [27]. All mice were weighed daily and monitored for clinical signs using a clinical scoring system. Blood was collected through tail vein nicking at day 1, 3, and 7 after administration, and serum samples were kept at −80 °C until analysis. Renal function (serum creatinine; Cr) and liver function (serum alanine transaminase; ALT) were measured by colorimetric assays using QuantiChromTM (DICT-500, BioAssay, Hayward, CA, USA) and EnzyChrom (EALT-100, BioAssay), respectively [26].

### 2.11 Ligand-binding to SARS-CoV-2 3CL^pro^

This study used SARS-CoV-2 3CL^pro^, 6M2N.pdb [28], as a protein receptor for molecular docking. The 3D protein structure in dimeric form was prepared as described previously [29]. The AM and GM chemical structures were constructed by Gaussview 6.0.16 program and subsequently optimized at the Density functional theory (DFT)/B3LYP level of theory with a 6-31G* basis set using the guassian16 program [30]. The empirical force field parameters and partial atomic charges were created according to the standard procedure [31]. The molecular parameters were generated using the PARMCHK2 module implemented in AmberTool 21 based on the general AMBER force field version 2 (GAFF2) [32]. The AMBER ff19SB force field was used for protein [33]. The AM and GM were docked into the active site of SARS-CoV-2 3CL^pro^ using AutoDock Vina 1.2.1 [34], according to the previous work [25].

The ligand/3CL^pro^ complex with the best molecular docking result was selected to examine the ligand-binding binding affinity and stability by performing the molecular dynamics (MD) simulation for 500 ns using AMBER 20 [32]. Details for MD simulation were performed as explained earlier [29]. A whole MD trajectory was analyzed in terms of root mean square deviation (RMSD) and radius of gyration (Rg) to evaluate the structural stability of each system. The last 100 ns-trajectories were chosen to calculate the principal component analysis (PCA) to explore the dynamical protein structure using CPPTRAJ module [35] in AMBER20.

### 2.12 Network-based target identification

The dataset of potential protein targets for AM and GM was predicted using the Chemical Similarity Ensemble Approach (SEA) [36,37]. Compounds with similar structures to AM and GM were identified through molecular fingerprint analysis, and their scores were determined using the Tanimoto algorithm [38]. The interactome of SARS-CoV-2 and host proteins was sourced from the CovInter database [39]. The data was then analyzed and visualized as a network using Cytoscape software [40]. In this network, nodes represent proteins associated with each compound, and edges depict Tanimoto scores ranging from 0 to 1—where a higher score signified a closer match, and the score number 1 was ideally the best match.

## 3 Result

### 3.1 Cytotoxicities and efficacies of AM and GM demonstrated antiviral potentials

**Fig. 1:**
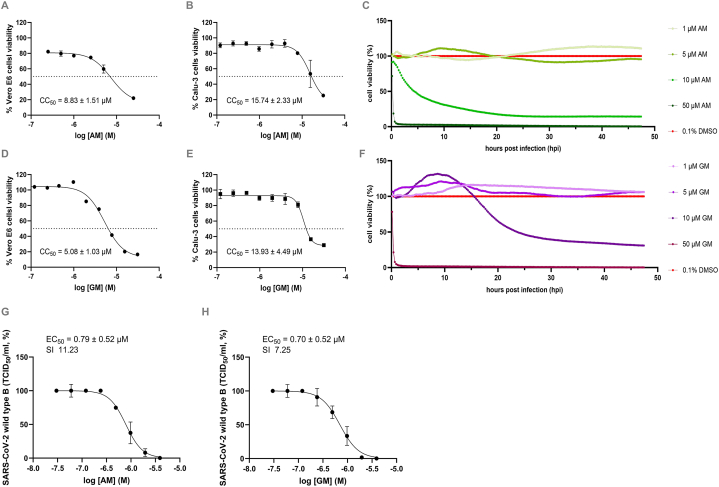
The cytotoxicity of **A-C)** AM and **D-F)** GM in mitochondrial toxicity and real-time cellular attachment assays. Various concentrations of AM and GM were incubated with **A,D)** Vero E6 and **B,E)** Calu-3 cells for 24 h before analyzing the mitochondrial toxicity by MTS reagent, respectively. CC_50_ were calculated from nonlinear regression and shown in means and SEM of three independent experiments. **C,F)** AM and GM at 1, 5, 10, and 50 μM were measured for 48 h cellular attachment by real-time cell analyzer. 0.1 % DMSO represented the 100 % cell viability. G-H) Effective concentration (EC_50_) and Selectivity indices (SI=CC_50_/EC_50_) of AM and GM against SARS-CoV-2 (wild type B) in Vero E6 cells. Results were reported as means and standard deviation (SD) of three independent experiments.

The cytotoxicity of AM and GM was accessed in Vero E6 and Calu-3 using classic mitochondrial toxicity and cell attachment by real-time measurement of cellular impedance (Fig. 1). CC_50_ by the mitochondrial toxicity assay at 24 h revealed that AM (Fig. 1A and B) and GM (Fig. 1D and E) were moderate to high toxicity against two mammalian cells, respectively. GM was slightly more toxic than AM in both cell lines, suggesting the contribution of the 7-OH moiety. Vero E6 was more susceptible and demonstrated apparent cytopathic effects (CPE) than Calu-3 cells; therefore, it was chosen for a further study of real-time cell analysis (RTCA). In cell-based kinetic profiling, AM (Fig. 1C) and GM (Fig. 1F) at 1, 5, 10, and 50 μM were incubated with Vero E6 cells for 48 h, and cellular detachment was detected by loss of impedance signal normalized by cells incubated with 0.1 % DMSO. The results showed that treatment with 1 and 5 μM AM and GM did not cause cellular toxicity and detachment within 48 h, while 10 and 50 μM AM and GM were cytotoxic and induced cellular detachment from 0 to 24 h after incubation. GM (1, 5, and 10 μM) obviously promoted cellular impedance signals in a dose-dependent manner at 0–24 h before returning to baseline or starting detachment at 24–28 h. The intermittent promotion could be the result of hydropic degeneration or cell swelling rather than proliferation. Moreover, cells were abruptly detached when incubated with 50 μM AM and GM, confirming the previous CC_50_ results. Note that cells incubated with 1 and 5 μM AM and GM maintained attachment throughout the study despite the CC_50_ of GM at 5.08 ± 1.03 μM (Fig. 1D). We speculated that AM and GM and 5 μM might intermittently induce reversible mitochondrial toxicity in Vero E6, but cells were still viable and fully attached to the surface detected by the impedance. Therefore, the concentrations taken for further studies should not exceed 5 μM. The effective concentration (EC_50_) of AM and GM against SARS-CoV-2 (wild type B) was described in submicromolar levels at 0.79 ± 0.52 μM and 0.70 ± 0.52 μM, respectively (Fig. 1G and H). Selectivity indices (SI=CC_50_/EC_50_) revealed that GM might be slightly more potent than AM as an antiviral agent.

### 3.2 The AM and GM inhibition was unlikely at the receptor-mediated binding, viral neutralization, or internalization

**Fig. 2:**
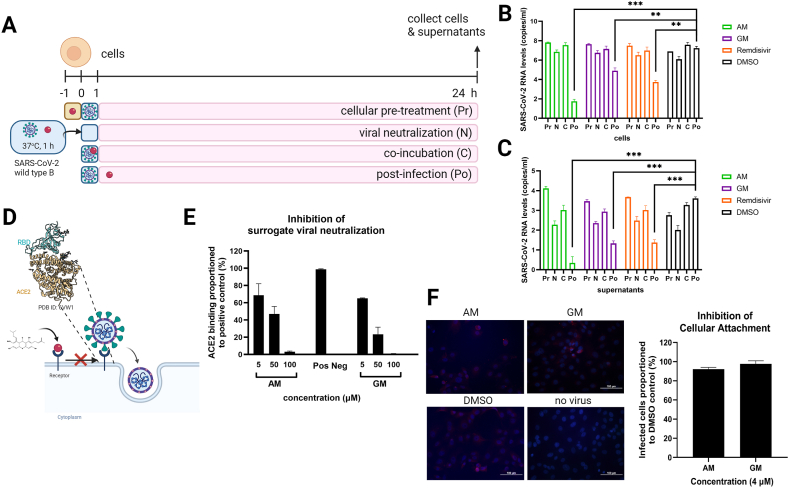
The binding inhibition analysis A) A scheme described the attachment inhibition protocols of 4 μM AM and GM with virus-infected cells under various conditions, and B) cell lysate and C) supernatants were harvested for viral RNA. Results were the means and standard deviation of two independent experiments. D) A scheme described RBD-ACE2 specific binding prior to inducing endocytosis. E) The surrogate neutralization test characterized the RBD/ACE2 binding efficiencies in the presence of AM and GM in a dose-dependent manner. Pos and neg represented positive and negative controls provided by the sVNT kit. F) An immunofluorescent assay characterized 4 μM AM- and GM-treated SARS-CoV-2-infected cells during attachment and compared with DMSO-treated cells. Results were the means and standard deviation of two independent experiments.

Next, we investigate the critical steps of viral replication mainly affected by the compounds (Fig. 2A–F). First, AM and GM were incubated with cells under various conditions, which are cellular pretreatment, viral neutralization, co-incubation, and post-infection (Fig. 2A) for 24 h before cell and supernatants to quantify viral RNA (Fig. 2B and C). AM and GM showed the strongest inhibition after infection (Fig. 2B and C), suggesting that the target should be located within the cells and should be a key factor for viral replication. AM and GM under other conditions did not show significant viral inhibition, suggesting that cellular receptor binding, viral neutralization, and internalization should not be the critical steps targeted by the compounds. Note that the inhibition of AM and GM was similar to those of another positive control in this experiment. Remdesivir is a nucleoside analog that inhibits viral replication by targeting RNA-dependent RNA polymerase (RdRp) of Coronaviruses. Furthermore, viral attachment requires specific bindings between the receptor-binding domain (RBD) of the viral spike and the ACE2 receptor (Fig. 2D). The results of the surrogated viral neutralization assay confirmed that the binding could not be the main target of AM and GM (Fig. 2E) as the concentration of AM and GM required to neutralize the binding of RBD-ACE2 was between 5 and 50 μM. Furthermore, inhibition of cell-based attachment was also evaluated by immunofluorescence. The 4 μM AM and GM were incubated with SARS-CoV-2 during 1 h of infection. The cells were washed and incubated for 6 h without the compound. Immunofluorescence depicting SARS-CoV-2 early translation (3CL^pro^) and the cellular nucleus were analyzed as a number of infected cells (Fig. 2F, Supplementary Fig. S1). The number of infected cells in AM- and GM-treated cells was insignificantly different from the DMSO-treated infected cells. Therefore, it was unlikely that AM and GM would primarily target viral attachment or ACE2 receptor-mediated binding and endocytosis.

### 3.3 The AM and GM inhibited 3CL^pro^ and N expressions in SARS-CoV-2-infected cells but the target was unlikely the 3CL^pro^ activity

**Fig. 3:**
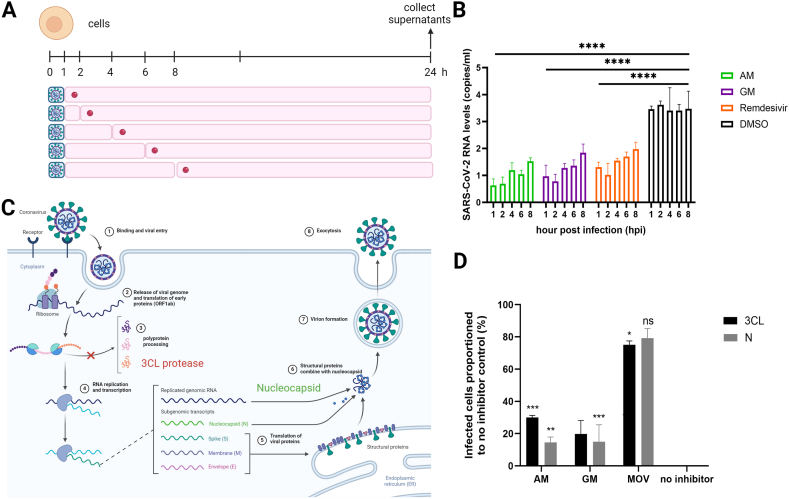
The translation and replication inhibition analysis A) A scheme described the time-of-addition study of 4 μM AM and GM with virus-infected cells and B) supernatants were harvested for analysis of viral RNA. The results were the means and standard deviation of two independent experiments. C) A scheme displayed 3CL^pro^ and N proteins expressing as early and late transcripts, respectively. D) Immunofluorescent signals of 3CL^pro^ and N proteins in 4 μM AM and GM treatment at 16 h proportionated to the no inhibition control. * = p-value <0.05 and ** = p-value <0.01.

Next, we explore which time-point after infection is critical for effective viral inhibition (Fig. 3A and B). AM and GM were then added 1, 2, 4, 6, and 8 h after the infection. The results showed that the strongest inhibition occurred at the earliest time-points. The inhibition became less efficient at later time points, suggesting that the inhibition occurred immediately after internalization. The inhibitory profiles of AM and GM were similar to those of remdesivir. Furthermore, the inhibition of all compounds was still significantly decreased at 8 h after infection compared to DMSO treatment. Previous articles suggested that viral translation occurred within 90 min after infection [41] and replaced host transcripts within 8 h after infection [42]. We then conclude that the translation/replication could be the critical step targeted by the compounds.

We further explored whether AM and GM inhibited SARS-CoV-2-infected cells using immunofluorescence against 3CL^pro^ and N expression (Fig. 3C and D, Supplementary Fig. S2). The results showed that the viral expressions of 3CL^pro^ and N in AM-, and GM-treated cells were significantly decreased at 16 h after infection, compared with the no inhibitor control (Fig. 3D, Supplementary S2). Molnupiravir, another anti-SARS-CoV-2 nucleoside analog, was a positive inhibitor control. Note that inhibition was more prominent in the N protein or late expression (Fig. 3C and D). The higher 3CL^pro^ signal could result from the accumulation of early transcripts. Therefore, AM and GM similarly showed translation inhibition profiles in both early and late viral proteins. It was likely that AM and GM would inhibit the viral translation and replication.

**Fig. 4:**
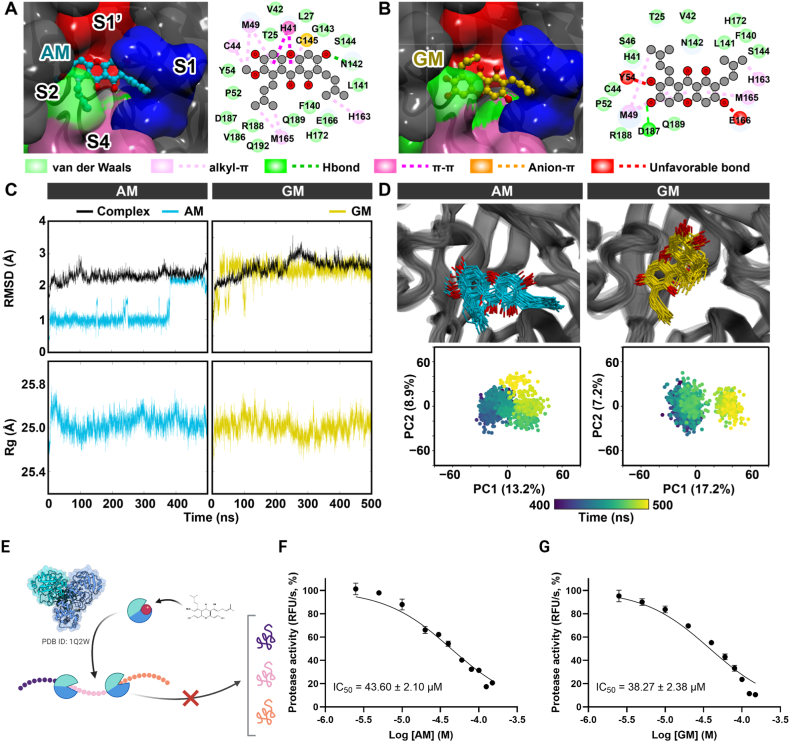
Analysis of SARS-CoV-2 3CL^pro^ as a potential target A-B) Molecular docking and C-D) MD trajectory analysis of AM and GM in complex with SARS-CoV-2 3CL^pro^ at the active site. E) A scheme described an *in vitro* protease activity assay and F-G) the inhibitory concentrations of the 3CL^pro^ activities. Data were means and standard deviation of triplicate results.

Previous *in silico* reports suggested that a SARS-CoV-2 3CL^pro^ protein could be a primary molecular target of mangostins [13,14]. Molecular _docking and MD simulations were used to explore AM and GM binding modes at the active 3CL^pro^ active site. Both AM and GM exhibited hydrophobic interactions (anion-π and π-π) with the catalytic dyad H41 and C145, producing binding interaction energies of 8.0 and −8.6 kcal/mol, respectively (Fig. 4A and B). The methylpentene insertion of AM into the S1 and S2 subpockets facilitated hydrophobic interactions with the M165 and H163 residues. In contrast, GM bound to the S1 and S1′ subpockets and formed alkyl-π interactions with M49 and H163. Both systems featured one hydrogen bond, although GM exhibited two unfavorable bonds towards Y54 and E166. These could reflect the slightly low inhibitory effect of GM on SARS-CoV-2. A 500-ns MD simulation showed fluctuating ligand binding stability based on the RMSD and Rg analysis (Fig. 4C). The AM binding stability is relatively steady until 380 ns, and then rearranged to another conformation in the last 120 ns. Meanwhile, the binding stability was worse in the GM system, which exhibited early instability (150–200 ns) and continued to fluctuate slightly over the simulation time. The dynamic behavior of complexes was depicted by the Cartesian coordinates of the first two principal components in PCA (PC1 and PC2, Fig. 4D) that showed the shared dynamical patterns during 400–500 ns. In particular, GM showed two subgroups, but AM showed only one, indicating that AM has greater stability. Despite slight instability in the binding pattern during MD simulation, both AM and GM could effectively restrict the protein motion, stabilizing the conformation at the 3CL^pro^ catalytic site, suggesting that 3CL^pro^ could be a target for AM and GM, but the binding patterns exhibited subtle differences. Similarly, an *in vitro* protease assay previously described [25] (Fig. 4E) showed that the IC_50_ values of AM and GM were 43.60 ± 2.10 μM, and 38.27 ± 2.38 μM, respectively (Fig. 4F and G). The results of the *in vitro* enzymatic assay did indeed support the *in silico* studies that 3CL^pro^ could be a potential target for AM and 10.13039/100002463GM. However, the level of discrepancies between IC_50_s (Fig. 4F and G) and EC_50_ (Fig. 1G and H) suggested that 3CL^pro^ was unlikely the major target. Additional targets involved in viral translation and replication should be expected.

**Fig. 5:**
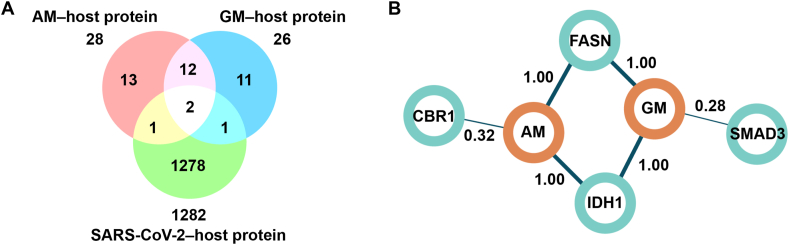
(A) Euler diagram illustrating proteins associated with AM, GM, and host proteins relevant to SARS-CoV-2 replication, and (B) identification of potential host protein targets for SARS-CoV-2 replication in AM and GM via network-based target identification. The numbers on each edge represent the Tanimoto coefficient, ranging from 0 to 1, with higher values indicating greater molecular structural similarity.

To explore alternative targets involved in viral translation and replication, we employed a network-based strategy [26], combining the SARS-CoV-2 host and compound-protein interaction networks [39](Fig. 5A). Using the Chemical Similarity Ensemble Approach (SEA) [37], we identified 28 and 26 potential protein targets for AM and GM, respectively. Both AM and GM interacted with type 1 tyrosine protein phosphatase non-receptor (PTN1), fatty acid synthase (FASN), and isocitrate dehydrogenase (IDH1). In particular, FASN and IDH1 exhibit links to the SARS-CoV-2 host protein interactome, suggesting essential roles in the context of SARS-CoV-2 infection (Fig. 5B). FASN is a key rate-limiting enzyme in the lipid synthesis pathway and the knockdown of FASN markedly reduced SARS-CoV-2 infection [43]. Recently, a report suggested that AM inhibited FASN expression and activity, thus reducing intracellular fatty acid accumulation [44]. Additionally, fatty acid synthesis is necessary for lipid remodeling and spherule formation for SARS-CoV-2 viral replication [45]. Furthermore, a lipid-lowering drug, orlistat, decreased the levels of the viral load in the lung, reduced lung pathology, and increased mouse survival [43]. Therefore, it was likely that AM would inhibit the viral replication via FASN in the fatty acid biosynthesis pathway, as shown in the KEGG pathway database (accession number: ec00061 [46]).

IDH1 catalyzes the decarboxylation of oxaloacetate to α-ketoglutarate and utilizes nicotinamide adenine dinucleotide phosphate (NADP+) as a cofactor, generating NADPH during catalysis. However, the IDH1 enzyme resides in the cytosol and does not participate in a tricarboxylic acid (TCA) cycle. Instead, IDH1 mainly contributed to generating NADPH to counteract reactive oxygen species induced by intrinsic metabolism and other cellular stressors. AM competitively inhibited the IDH1-R132H [47], which is an IDH1 mutant converting alpha-ketoglutarate to D-2-hydroxyglutarate (D2HG). Accumulating D2HG-induced global DNA hypermethylation and IDH1 mutation is related to neurological and hematological neoplasms [48]. Moreover, D2HG enhances the binding of DNMT1 to IRF3/7 promoters, thus down-regulated IRF3/7 and impaired type I IFN response [49]. However, the IDH1-mutated gene was uncommon in the general population. Further chemical structure similarity analysis revealed a similar structure of AM associated with carbonyl reductase 1 (CBR1), while GM linked to the mothers against decapentaplegic homolog 3 (SMAD3). CBR1 is an NADPH-dependent oxidoreductase responsible for metabolizing toxic quinones [50] and preventing cellular oxidative stress [51]. Mothers against decapentaplegic homolog 3 (SMAD3) is activated by a transforming growth factor beta (TGF-β) signaling pathway and reduced a proto-oncogene, c-myc, expression. Therefore, only CBR1 could be relevant to the virus-induced cellular stress, suggesting that only AM might also be its inhibitor.

The network-based target identification proposed that AM and GM could potentially target FASN and thereby inhibit SARS-CoV-2-infected cells through interference with lipid remodeling. Additionally, another potential target indirectly associated with the compound-induced viral inhibition might exist.

### 3.4 Efficacy of AM and GM as potential inhibitors of SARS-CoV-2 variants

**Fig. 6:**
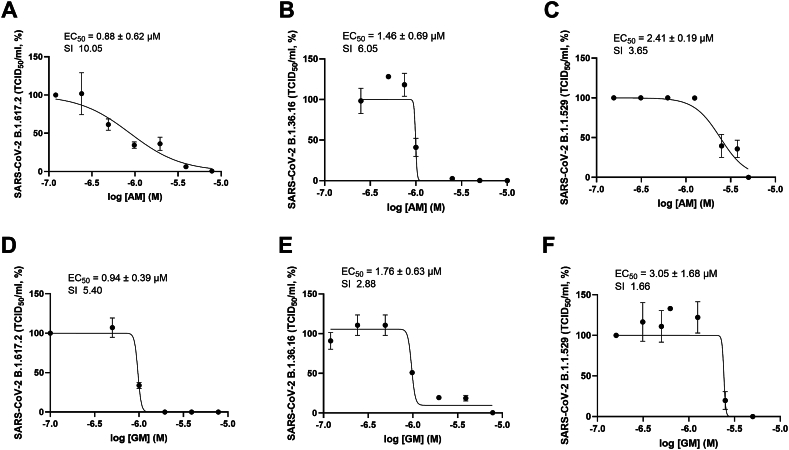
Effective concentration (EC_50_) and Selectivity indices (SI=CC_50_/EC_50_) of A-C) AM and D-F) GM against SARS-CoV-2 variants; B1.617.2, B.1.36.16, B.1.1.529 in Vero E6 cells. Results were reported as means and standard deviation (SD) of three independent experiments.

SARS-CoV-2 variants and escape mutants have continuously evolved to evade immunological responses and increase viral fitness in the human population. We chose three variants of SARS-CoV-2, including B.1.617.2, B.1.36.16, and B.1.1.529 as representatives of a variant of concern (delta), a 2021 non-VOC 2021, and the 2022 variant of concern (omicron), respectively (Fig. 6A–F). The results showed that AM and GM EC_50_ reduced their efficacies in all variants, thus the SI became 1.66–10.05. Moreover, AM inhibited the variants more effectively than GM, in contrast to the wild-type inhibition (Fig. 1G and H). AM and GM were the least effective in the latest variant, B.1.1.529, with the EC_50_ of 2.41 ± 0.19 and 3.05 ± 1.68 μM, respectively. This observation implied that the molecular target or pathway could be significantly exploited in the wild type B, but not in the B.1.1.529 variant.

### 3.5 In vivo toxicity

**Fig. 7:**
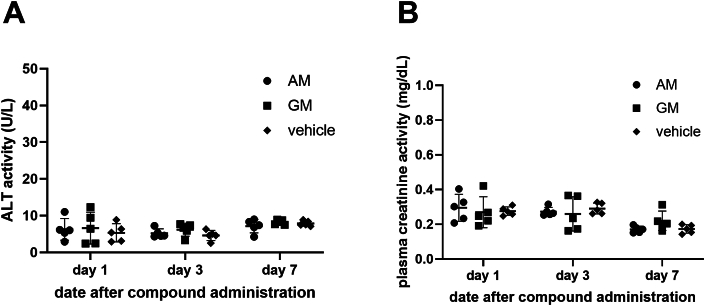
*In vivo* toxicity study of 25 mg/kg AM and GM in 1, 3, 7 day after administration. Mice were intraperitoneally administered with a single dose of AM, GM, or vehicle, and blood were taken for analysis of alanine transminase and serum creatinine levels. No significant difference were observed among groups.

Toxicity was preliminarily assessed in immunocompetent mice using a single dose of 25 mg/kg AM and GM intraperitoneally. In this experiment, the 25 mg/kg concentration was chosen according to the LD_50_ in BALB/C in the previous report [16]. The crude methanolic extract (CME) containing 25.19 % AM showed BALB/c LD_50_ at 200 mg/kg, inferring that AM LD_50_ may not exceed 50 mg/kg. The 25 mg/kg dose was expected to be a high, non-toxic dose for AM. No previous report on GM LD_50_ was discovered; therefore, we chose the same concentration with AM. The results did not show significantly elevated alanine transaminase (ALT) or serum creatinine, suggesting no hepatorenal toxicity on days 1, 3, and 7 after injection (Fig. 7A and B). Therefore, we concluded that AM and GM did not show any acute toxicity in mice. Further investigation would include pharmacokinetics and antiviral efficacy tests.

## 4 Discussion

This article described that AM and GM indeed inhibited SARS-CoV-2-infected cells with efficacies at submicromolar levels and selectivity indices at 11.23 and 7.25, respectively. Compounds mainly inhibited post-infection processes and suppressed viral replication (Fig. 3A and B) and protein expressions (Fig. 3C and D). Little inhibitory effect was found in attachment and internalization (Fig. 2). Moreover, their efficacy was diminished in three variants, with the underlying reasons remain unclear. In particular, CC_50_ values exceeding 10 μM indicated non-toxicity in RTCA assays, implying that viral inhibition probably did not stem from compound toxicity. Our CC_50_ of AM was similar to other studies at 10–20 μM to MDA-MB-231 and MCF-7 cells [52]. Therefore, we concluded that AM and GM inhibited the translation and replication.

The compounds were expected to interact with various molecules derived from the virus and the host, including 3CL^pro^, and ACE2. Among these targets, 3CL^pro^ attracted considerable attention in *in silico* studies. However, our enzymatic findings (IC_50_ at 43.60 ± 2.01 and 38.27 ± 2.38 μM; Fig. 4E and F) and docking simulations (Fig. 4A–D) indicated that 3CL^pro^ could serve as an adjunctive target, while the main target remained unidentified. Moreover, several factors supported the claim that 3CL^pro^ was unlikely to be the sole target. First, the 3CL^pro^ sequences were conserved among wild-type and variant strains; therefore, similar protease activities and inhibition of SARS-CoV-2 should be expected from all strains. However, we observed that the efficacies of the wild-type were superior to those of the mutants. Second, the observed disparities between the IC_50_ and EC_50_ of wild-type enzymes and infected cells, respectively, make it improbable that 3CL^pro^ acts as the main target. Third, our *in silico* approach suggested that hydrophobic interactions (anion-π and π-π) with the catalytic dyad (H41 and C145) fluctuated in a 500 ns MD simulation, altering the stability of the binding of the ligand. Therefore, we concluded that 3CL^pro^ could be an adjunctive target of AM and GM due to their unstable bindings.

An article suggested that the specific binding of ACE2 and ACE2-RBD could be a target [15]. We explored cell-based attachment, sVNT, and immunofluorescence and found that the pre, N, and co-that were all potential conditions involving ACE2 did not differ significantly from controls treated with DMSO. The IFA results (Fig. 2F) showed that 4 μM AM and GM could not inhibit viral entry and establish initial translation. Although sVNT showed a dose-dependent inhibition, the 50 % inhibition was ≥5 μM, which was likely to reach the cell-based CC_50_ result. Therefore, we concluded that ACE2-binding and internalization were less likely to contribute to the SARS-CoV-2 inhibition by AM and GM.

*In silico* approaches suggested that a fatty acid synthase (FASN) was probably a target of AM- and GM-inhibiting SARS-CoV-2 translation and replication (Fig. 4A and B). FASN chemical inhibitors or siRNAs were correlated with the reduction of viral replications in various strains of SARS-CoV-2, as well as other viruses; VSV, HSV-1, MHV68, dengue, chikungunya, HIV and HCV [[53], [54], [55], [56]]. Furthermore, AM inhibited FASN expression and activity [57], implying that FASN might be a potential target of AM and GM, reducing SARS-CoV-2 inhibition. FASN catalyzed long chain fatty acids (C16:0 palmitate) involved in lipid remodeling and droplet formation [53]. Furthermore, FASN was recruited to the DENV-infected cell replication site and was associated with an increasing rate of fatty acid biosynthesis [58]. Therefore, it was likely that AM would suppress FASN in cells, thus reducing viral replication as observed in this work. However, FASN was shown to be the target of all SARS-CoV-2 and two FASN inhibitors could inhibit various strains of SARS-CoV-2 infections, but no strain preference was discussed. Our results showed that AM and GM inhibited SARS-CoV-2 infections, most potently in the wild-type. Unanswered questions remained of why FASN was preferentially suppressed in wild-type SARS-CoV-2. We speculated that later variants were better adapted to mammalian cells and more efficiently upregulating FASN expression and activities. Therefore, higher concentrations of AM and GM would be required to inhibit the SARS-CoV-2 variants. Further investigations should include analysis of FASN expression levels in wild-type and GM-treated SARS-CoV-2 variants treated with AM and GM.

The toxicities were explored using cell and animal experiments. Based on the previous study [59], Vero E6 and Calu-3 cell lines were chosen for analysis. Calu-3, a human lung adenocarcinoma cell line, showed higher tolerance against the compounds at 15.74 and 13.93 μM. Furthermore, the additional cytotoxicity assay (real-time cell analysis, RTCA) measuring the electrical impedance of cell attachment suggested that the effective dose (5 μM) of AM and GM demonstrated the cell attachment pattern similar to that of the 0.1 % DMSO vehicle control (Fig. 2A and B). Therefore, we concluded that 5 μM AM and GM could inhibit SARS-CoV-2 infection (Fig. 1) without apparent cytotoxicities (Fig. 2). However, the CC_50_ in Vero E6 was below 10 μM which were narrow compared to other natural products (e.g. flavonoids). Similar AM toxicities have been described in DLD-1 cells from human colon cancer DLD-1 cells [60], human leukemia HL60 cells [61], chondrosarcoma [62], and melanoma cells [63], mainly by inducing apoptosis through various pathways. Obviously, mangostins, especially AM, were evidently cytotoxic to most cell lines. Therefore, further structural modifications for AM derivatives should be seriously considered in order to reduce the toxicity in parallel with increasing the efficacies.

Furthermore, an LD_50_ dose (25 mg/kg) administered intraperitoneally in a single dose was not toxic, as the ALT and Cr levels representing hepatorenal functions were within the normal range (Fig. 7). In contrast to cytotoxicity, several reports simultaneously agreed that the extracts and AM were generally safe and well tolerated in humans [64,65] and animals [[66], [67], [68], [69]]. Extensive pharmacokinetic studies were also performed with AM and mangosteen extracts. Furthermore, AM was abundant in mangosteen pericarp, considered an agricultural waste. Therefore, it was economically likely to scale up the extraction and experiment on the structural modification for higher antiviral efficacies and milder cytotoxicity.

## 5 Conclusion

The AM and GM effectively inhibited the wild-type SARS-CoV-2 infected cells but were attenuated in three mutants. The potential targets were still elusive. The 3CL^pro^ and ACE2 were likely adjunctive targets as their inhibitory concentrations largely discrepant with the cellular efficacies. There was no observed hepatorenal toxicity in mice.

## Ethics declarations

This study was reviewed and approved by the Institutional Review Board of Faculty of Medicine, Chulalongkorn University certified the protocol exemption for using a leftover specimen (COE 017/2021, IRB No. 297/64), according to the guidelines of the Declaration of Helsinki, and Chulalongkorn University Institutional Biosafety Committee (CU-IBC 003/2021). All experimental protocols were approved by the Institutional Animal Care and Use Committee of the Faculty of Medicine, Chulalongkorn University, Bangkok, Thailand (certificate number: 017/2565), based on the National Institutes of Health, USA's criteria for the use and treatment of laboratory animals. All methods are reported in accordance with ARRIVE guidelines (https://arriveguidelines.org) for the reporting of animal experiments.

## Data availability statement

The data that support the findings of this study are available on request from the corresponding author, [SB].

## CRediT authorship contribution statement

**Aphinya Suroengrit:** Writing – original draft, Project administration, Methodology, Investigation. **Van Cao:** Methodology, Investigation. **Patcharin Wilasluck:** Methodology, Investigation. **Peerapon Deetanya:** Methodology, Investigation. **Kittikhun Wangkanont:** Formal analysis, Data curation, Conceptualization. **Kowit Hengphasatporn:** Software, Investigation, Formal analysis, Data curation, Conceptualization. **Ryuhei Harada:** Software, Resources, Investigation. **Supakarn Chamni:** Resources, Conceptualization. **Asada Leelahavanichkul:** Supervision, Resources, Investigation. **Yasuteru Shigeta:** Supervision, Resources, Funding acquisition. **Thanyada Rungrotmongkol:** Writing – review & editing, Supervision, Resources, Conceptualization. **Supot Hannongbua:** Supervision, Resources, Conceptualization. **Warinthorn Chavasiri:** Writing – review & editing, Supervision, Resources, Investigation. **Supaporn Wacharapluesadee:** Supervision, Resources, Data curation. **Eakachai Prompetchara:** Resources, Methodology, Investigation. **Siwaporn Boonyasuppayakorn:** Writing – review & editing, Writing – original draft, Validation, Funding acquisition, Formal analysis, Data curation, Conceptualization.

## Declaration of competing interest

The authors declare that they have no known competing financial interests or personal relationships that could have appeared to influence the work reported in this paper.
