# Supplementary material for: Alpha and gamma mangostins inhibit wild-type B SARS-CoV-2 more effectively than the SARS-CoV-2 variants and the major target is unlikely the 3C-like protease
Source: Heliyon. 2024 May 27;10(11):e31987. doi: 10.1016/j.heliyon.2024.e31987 (PMC11168321; doi:10.1016/j.heliyon.2024.e31987)
Supplement: Multimedia component 1 [file mmc1.docx]

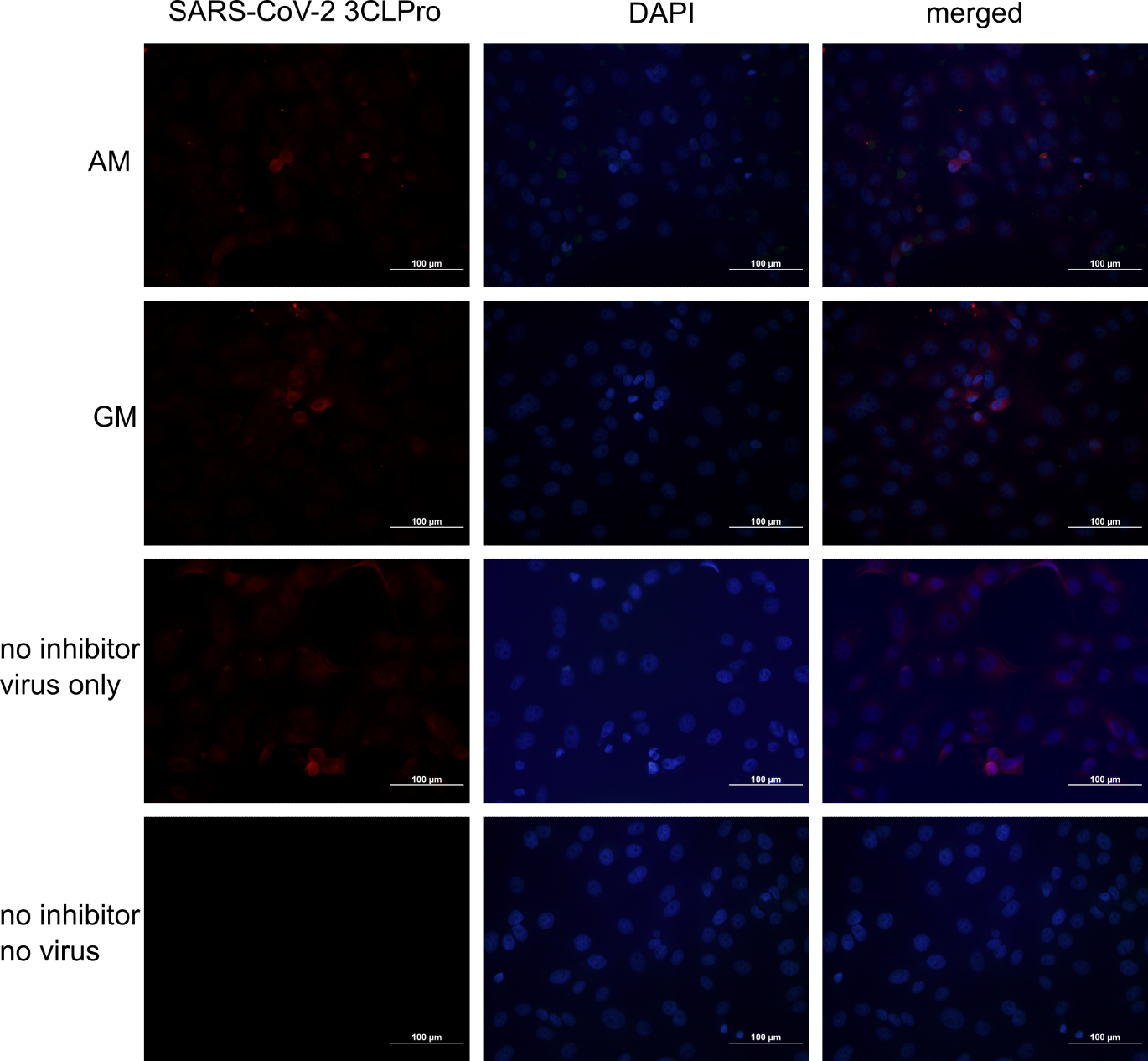


Fig. S1 Immunofluorescent assays of an early protein, 3CLpro, in the presence of 4 µM AM and GM during attachment of SARS-CoV-2 to the Vero E6 cells. Cells were fixed and stained at 6 h post-infection. A number of infected cells were quantified, compared with DMSO-treated infected cells, and reported in Fig. 2F.


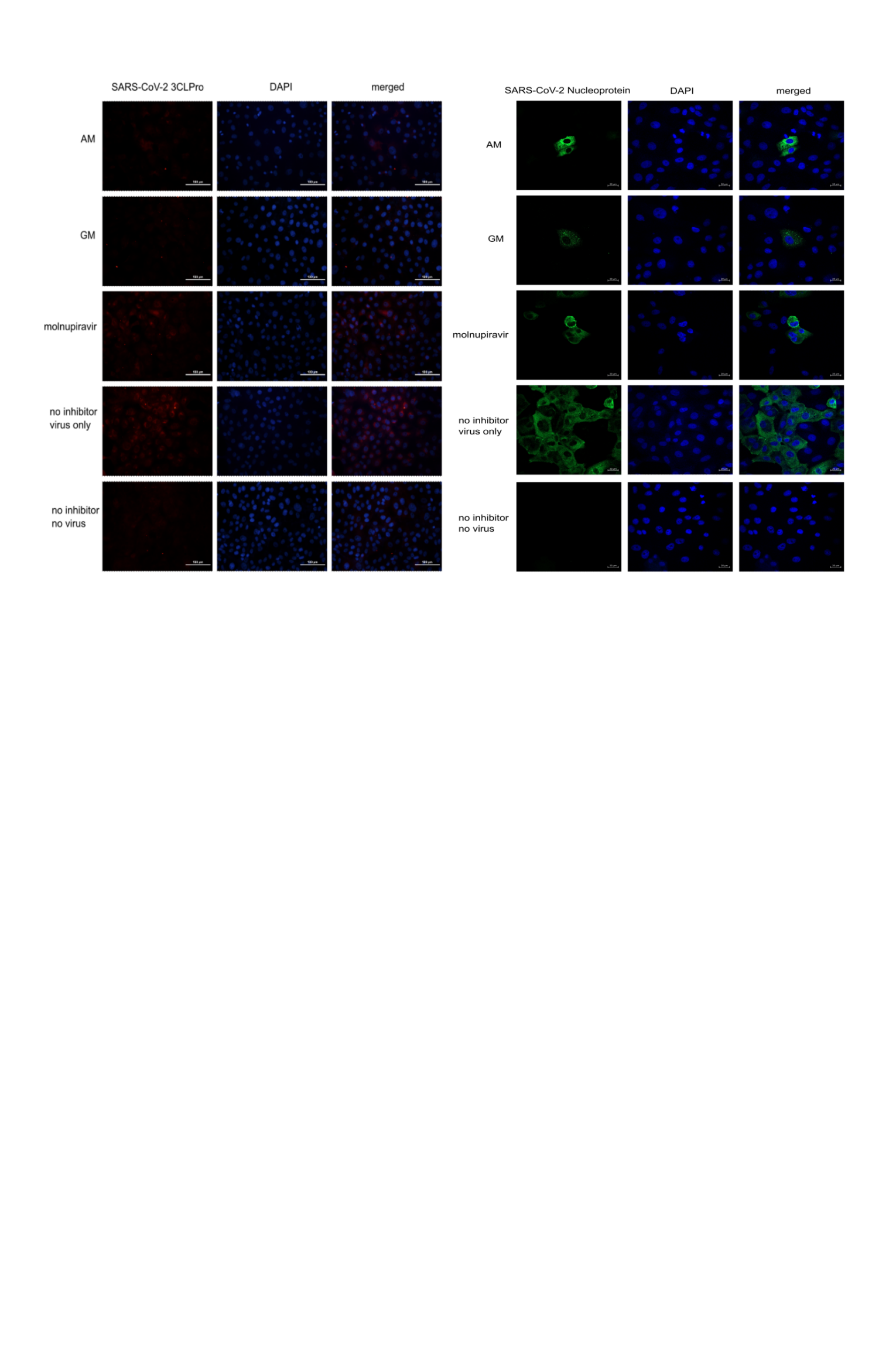
Fig. S2 Immunofluorescent assays of early and late, 3CLpro and N, proteins in the presence of 4 µM AM and GM after infection with SARS-CoV-2 to the Vero E6 cells. Cells were fixed and stained at 16 h post-infection. A number of infected cells were quantified proportionated to the no inhibition control and reported in Fig. 3D.
